# Supplementary material for: Eosinophils improve cardiac function after myocardial infarction
Source: Nat Commun. 2020 Dec 16;11:6396. doi: 10.1038/s41467-020-19297-5 (PMC7745020; doi:10.1038/s41467-020-19297-5)
Supplement: Supplementary file 1 — Supplementary Information [file 41467_2020_19297_MOESM1_ESM.pdf]

Supplementary Information

Eosinophils improve cardiac function after myocardial infarction

Jing Liu,[1,2]* Chongzhe Yang,[1]* Tianxiao Liu,[1,2]* Zhiyong Deng,[1]* Wenqian Fang,[1] Xian Zhang,[1] Jie Li,[1] Qin Huang,[1] Conglin Liu,[1] Yunzhe Wang,[1] Dafeng Yang,[1] Galina K. Sukhova,[1] Jes S. Lindholt,[3,4,5] Axel Diederichsen,[4,6] Lars M. Rasmussen,[4,7] Dazhu Li,[2] Gail Newton,[8] Francis W. *Luscinskas,*[8] Lijun Liu,[9] Peter Libby,[1] Jing Wang,[10] Junli Guo,[1,11] Guo-Ping Shi[1]

1. Department of Medicine, Brigham and Women's Hospital and Harvard Medical School, Boston, MA 02115, USA
2. Laboratory of Cardiovascular Immunology, Institute of Cardiology, Union Hospital, Tongji Medical College, Huazhong University of Science and Technology, Wuhan 430022, China
3. Department of Cardiothoracic and Vascular Surgery, Odense University Hospital, Odense, Denmark
4. Elitary Research Centre of personalised medicine in arterial disease (CIMA), Odense University Hospital, Odense, Denmark
5. Cardiovascular Research Unit, Viborg Hospital, Denmark
6. Department of Cardiology, Odense University Hospital, Odense, Denmark
7. Department of Clinical Biochemistry and Pharmacology, Odense University Hospital, Odense, Denmark
8. Department of Pathology, Brigham and Women's Hospital and Harvard Medical School, Boston, MA 02115, USA
9. Department of Biochemistry and Cancer Biology, College of Medicine and Life Sciences, University of Toledo, Toledo, OH 43614, USA
10. State Key Laboratory of Medical Molecular Biology, Institute of Basic Medical Sciences, Chinese Academy of Medical Sciences, Department of Pathophysiology, Peking Union Medical College, Beijing, China.
11. Key Laboratory of Emergency and Trauma of Ministry of Education & Research Unit of Island Emergency Medicine, Chinese Academy of Medical Sciences, Hainan Provincial Key Laboratory for Tropical Cardiovascular Diseases Research, The First Affiliated Hospital, Hainan Medical University, Haikou 571199, China

Supplementary Tables

Supplementary Table 1. Baseline information of patient with (Yes) and without (No) respective variables.

Variable	Yes		No		
	N	EOS (x10^9/L) Mean (SD)	N	EOS (x10^9/L) Mean (SD)	*P**
Age >=70 years	1626	0.213 (0.148)	3903	0.211 (0.157)	0.651
Current or former smoker	3708	0.218 (0.162)	1796	0.198 (0.135)	<0.001
Hypertension	2553	0.213 (0.141)	2977	0.210 (0.165)	0.415
Diabetes Mellitus	633	0.239 (0.178)	4897	0.208 (0.151)	<0.001
**Comorbidity**					
Previous stroke	411	0.219 (0.174)	5119	0.211 (0.153)	0.278
Previous AMI	345	0.261 (0.261)	5185	0.208 (0.144)	<0.001
Previous peripheral artery disease	138	0.215 (0.123)	5392	0.211 (0.155)	0.751
Abdominal aortic aneurysm	113	0.211 (0.123)	5417	0.211 (0.155)	0.905
Atrial fibrilation	775				
Previous coronary intervention	434	0.242 (0.237)	5096	0.209 (0.145)	<0.001
Heart valve replacement	71	0.226 (0.148)	5459	0.211 (0.154)	0.431
Chronic pulmonary obstructive disease	404	0.259 (0.209)	5126	0.207 (0.148)	<0.001
**Medication**					
Use of low dose aspirin	1257	0.229 (0.192)	4273	0.206 (0.141)	<0.001
Use of warfarin	397	0.212 (0.159)	5133	0.211 (0.154)	0.957
Use of Statin	1500	0.226 (0.183)	4030	0.206 (0.141)	<0.001
Use of ACE inhibitor or AT2-antagonists	1900	0.217 (0.163)	3630	0.208 (0.149)	0.061
Use of calcium blocker	1047	0.213 (0.138)	4433	0.211 (0.158)	0.737
Use of loop diuretics	251	0.250 (0.183)	5279	0.209 (0.152)	<0.001
Use of beta-agonist	173	0.295 (0.232)	5357	0.209 (0.150)	<0.001
Use of inhalation glucocorticoid	306	0.278 (0.291)	5224	0.207 (0.141)	<0.001
Use of oral glucocorticoid	104	0.195 (0.193)	5426	0.215 (0.153)	0.292
Use of NSAID	182	0.213 (0.161)	5348	0.211 (0.154)	0.862
**Symptoms**					
Chest pain	934	0.235 (0.210)	4596	0.207 (0.14)	<0.001
Dypnoe	990	0.234 (0.206)	4530	0.206 (0.140)	<0.001
**Body mass index**					
<18.5	18	0.180 (0.149)			
18.5-25	1198	0.200 (0.148)			
25-30	2638	0.213 (0.167)			
>30	1665	0.216 (0.136)			0.025
**Mobility score (Eur-Qol-5D)**					
No problems	4640	0.208 (0.150)			
Some problems (Eur-Qol-5D)	880	0.230 (0.176)			
Bed bound	4	0.230 (0.111)			0.001
**NYHA classification score**					
NYHA class 1	4871	0.207 (0.150)			
NYHA class 2	456	0.239 (0.173)			
NYHA class 3	176	0.250 (0.189)			
NYHA class 4	12	0.366 (0.361)			<0.001

Abbreviations: NSAID–nonsteroidal anti-inflammatory drug; NYHA– The New York Heart Association. *Student t test.

Supplementary Table 2. Multivariate logistic regression analysis of previous AMI dependent variables.

Dependent variable	B	S.E.	*P*	OR	95% C.I. Lower	95% C.I. Upper
EOS count ($x10^9$/L)	0.988	0.357	0.006	2.685	1.335	5.401
BMI group	0.180	0.091	0.049	1.197	1.001	1.432
Former of current smoking	0.363	0.157	0.020	1.438	1.058	1.954
COPD	0.051	0.206	0.804	1.052	0.703	1.575
Diabetes mellitus	-0.163	0.160	0.306	0.849	0.621	1.161
Use of low dose aspirin	2.773	0.182	0.000	16.005	11.193	22.886
Use of statin	1.638	0.161	0.000	5.145	3.755	7.050
Constant	-6.004	0.299	0.000	0.002		

Supplementary Table 3. Multivariate logistic regression analysis of NYHA classification dependent variables.

Dependent variable	Unstandardized coefficient			95% C.I. for B		Correlations	
	B	Std error	*P*	Lower	Upper	Zero-Order	Partial
(Constant)	-0.100	0.020	0.000	-0.139	-0.061		
EOS count ($x10^9$/L)	0.102	0.036	0.005	0.030	0.173	0.082	0.038
BMI group	0.062	0.008	0.000	0.046	0.077	0.115	0.105
Former or current smoking	0.037	0.012	0.003	0.013	0.060	0.108	0.041
COPD	0.636	0.022	0.000	0.593	0.679	0.382	0.366
Diabetes mellitus	0.067	0.018	0.000	0.030	0.103	0.101	0.049
Use of low dose aspirin	0.089	0.015	0.000	0.059	0.118	0.142	0.080
Use of statin	0.018	0.015	0.204	-0.010	0.047	0.103	0.017

Supplementary Table 4. RT-PCR primer sequences.

Gene	Forward primers	Reverse primers
β-actin	5'-GTGACGTTGACATCCGTAAAGA-3'	5'-GCCGGACTCATCGTACTCC-3'
Icam1	5'-GTGATGCTCAGGTATCCATCCA-3'	5'-CACAGTTCTCAAAGCACAGCG-3'
Vcam1	5'-AGTTGGGGATTCGGTTGTTCT-3'	5'-CCCCTCATTCCTTACCACCC-3'
E-selectin	5'-ATGCCTCGCGCTTTCTCTC-3'	5'-GTAGTCCCGCTGACAGTATGC-3'
Ccr3	5'-TCGAGCCCGAACTGTGACT-3'	5'-CCTCTGGATAGCGAGGACTG-3'
Eotaxin	5'-GAATCACCAACAACAGATGCA-3'	5'-ATCCTGGACCCACTTCTTCTT-3'
Eotaxin-2	5'-TCTTGCTGCACGTCCTTTATT-3'	5'-GCATCCAGTTTTTGTATGTGCC-3'
Eotaxin-3	5'-TTCTTCGATTTGGGTCTCCTTG-3'	5'-GTGCAGCTCTTGTCGGTGAA-3'
Rantes	5'-TTTGCCTACCTCTCCCTCG-3'	5'-CGACTGCAAGATTGGAGCACT-3'
Mip1α	5'-TGTACCATGACACTCTGCAAC-3'	5'-CAACGATGAATTGGCGTGGAA-3'
Mip1β	5'-TTCCTGCTGTTTCTCTTACACCT-3'	5'-CTGTCTGCCTCTTTTGGTCAG-3'
Mcp1	5'-TTAAAAACCTGGATCGGAACCAA-3'	5'-GCATTAGCTTCAGATTTACGGGT-3'
Mcp2	5'-CTGGGCCAGATAAGGCTCC-3'	5'-CATGGGGCACTGGATATTGTT-3'
Mcp3	5'-CCTCTGGATAGCGAGGACTG-3'	5'-ACACCGACTACTGGTGATCCT-3'
Cxcl1	5'-ACTGCACCCAAACCGAAGTC-3'	5'-TGGGGACACCTTTTAGCATCTT-3'
Cxcl2	5'-CCAACCACCAGGCTACAGG-3'	5'-GCGTCACACTCAAGCTCTG-3'
Cxcl3	5'-CAGCCACACTCCAGCCTA-3'	5'-CACAACAGCCCCTGTAGC-3'
Cxcl5	5'-GTTCCATCTCGCCATTCATGC-3'	5'-GCGGCTATGACTGAGGAAGG-3'
Cxcl7	5'-CTCAGACCTACATCGTCCTGC-3'	5'-GTGGCTATCACTTCCACATCAG-3'
Collagen1	5'-GCTCCTCTTAGGGGCCACT-3'	5'-ATTGGGGACCCTTAGGCCAT-3'
Collagen3	5'-CCTGGCTCAAATGGCTCAC-3'	5'-GACCTCGTGTTCCGGGTAT-3'

Supplementary Figures

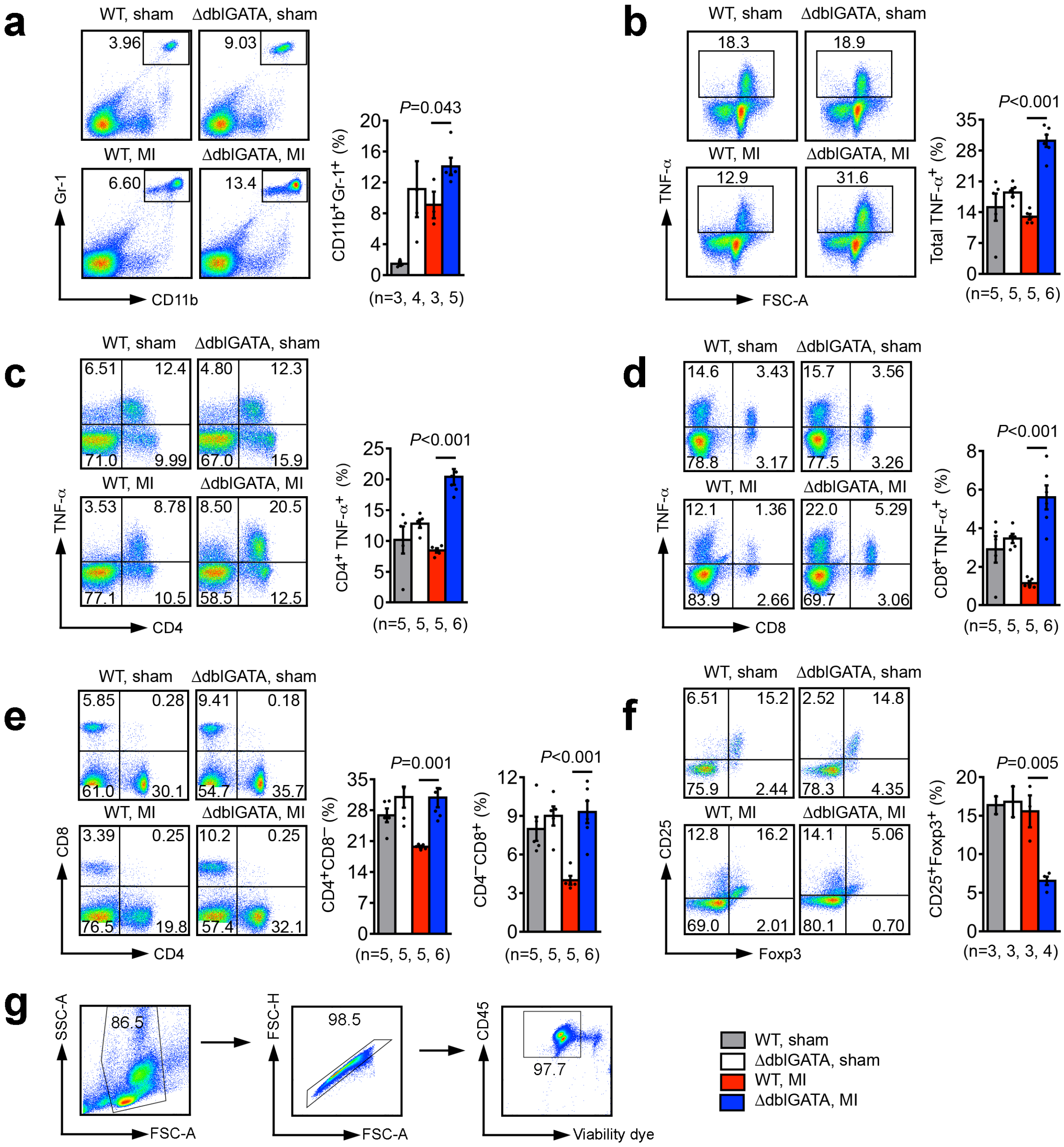

Supplementary Fig. 1. FACS analysis of splenic CD45^{+} immune cells from different groups of mice as indicated at 1-month post-MI. **a.** CD11b^{+}Gr-1^{+} neutrophils. **b.** Total TNF-α^{+} cells. **c.** CD4^{+}TNF-α^{+} T cells. **d.** CD8^{+}TNF-α^{+} T cells. **e.** CD4^{+}CD8^{-} and CD4^{-}CD8^{+} T cells. **f.** CD4^{+}CD25^{+}Foxp3^{+} Tregs. **g.** CD45^{+} immune cell FACS gating strategy. Representative FACS images are shown to the left of each panel. Data are mean±SEM. The number of mice in each group and *P* values are indicated, one-way ANOVA test.

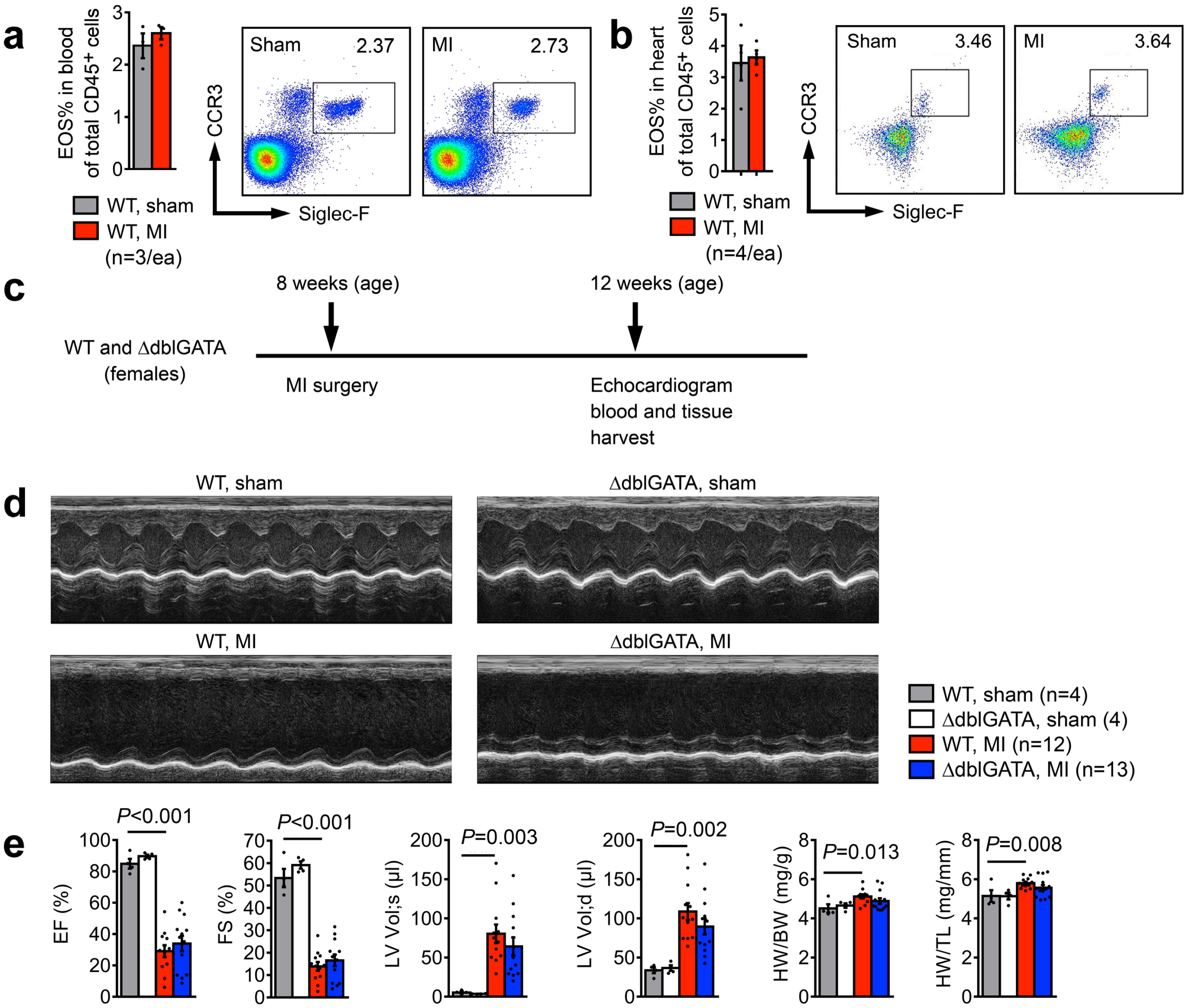

Supplementary Fig. 2. EOS deficiency does not affect cardiac functions post-MI in female mice. **a/b.** FACS analysis of blood (**a**) and heart (**b**) EOS in female mice at 1day post-MI or sham. FACS gating strategy is shown in **Fig. 1c** in the main text. **c.** MI surgery scheme. **d.** Representative LV M-mode echocardiography images at 1-month post-MI. **e.** Cardiac functions at 1-month post-MI: EF, FS, LV Vol;d, LV Vol;s, HW/BW, and HW/TL of different mice as indicated. Data are mean±SEM. The number of mice in each group and *P* values are indicated, non-parametric Mann-Whitney *U* test (**a/b**) or one-way ANOVA test (**e**).

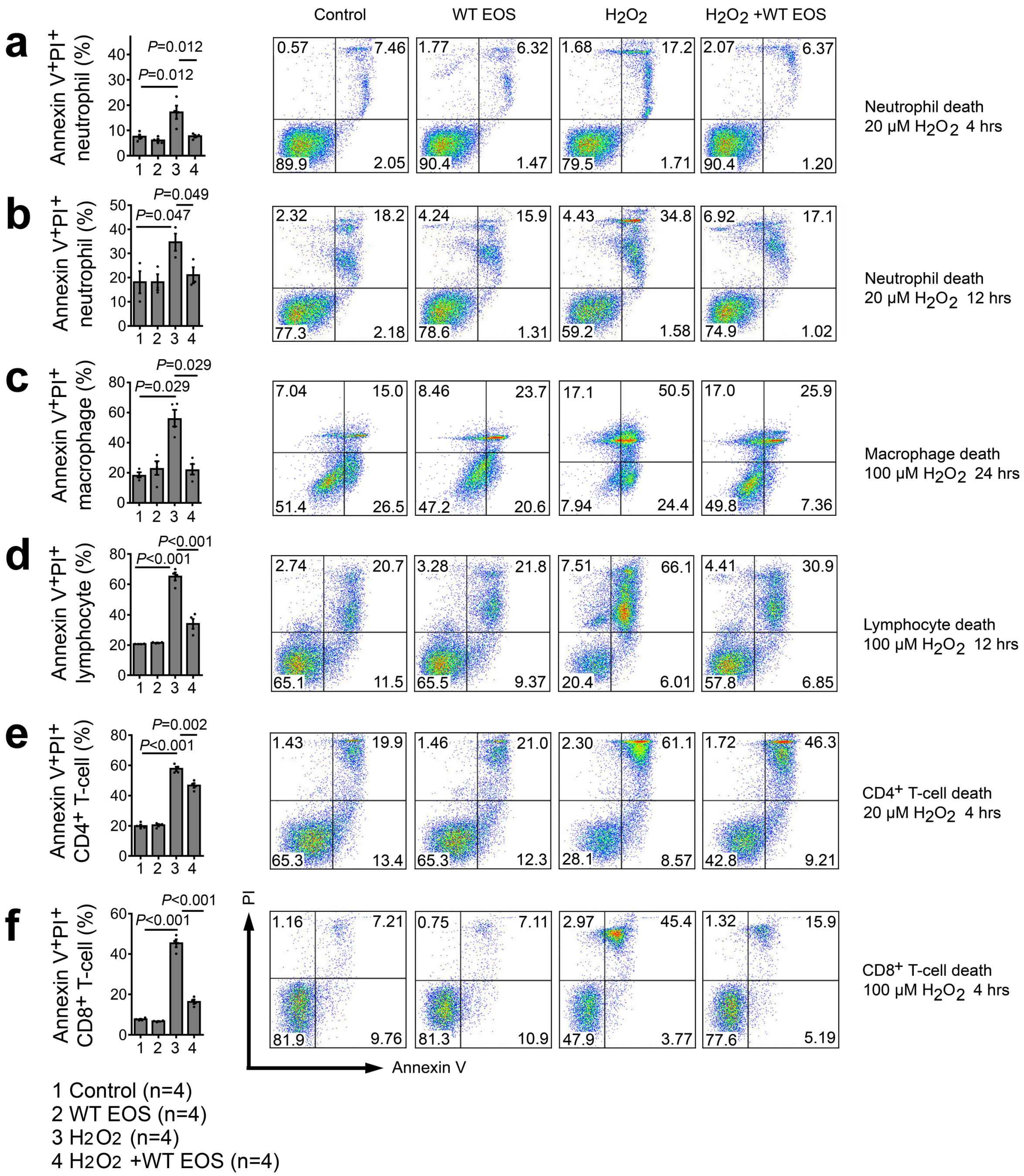

Supplementary Fig. 3. WT EOS activity in protecting immune cells from apoptosis. FACS detected H_2O_2-induced Annexin V$^+$PI$^+$ late apoptotic neutrophils (**a/b**), macrophages **(c)**, splenic lymphocytes **(d)**, CD4$^+$ T cells **(e),** and CD8$^+$ T cells **(f)** after cells were treated with or without 20 µM H_2O_2 and WT EOS lysate equivalent to10^6 EOS/ml at indicated hrs. Data are mean±SEM. The number of mice in each group and *P* values are indicated, one-way ANOVA test.

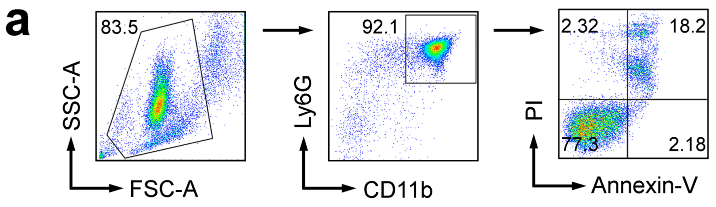

Bone-marrow neutrophil apoptosis gating strategy for Fig. S3a and S3b.

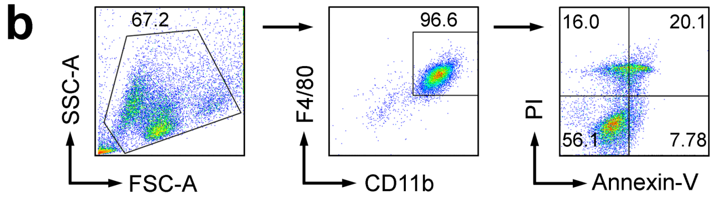

Macrophage apoptosis gating strategy for Fig. S3c.

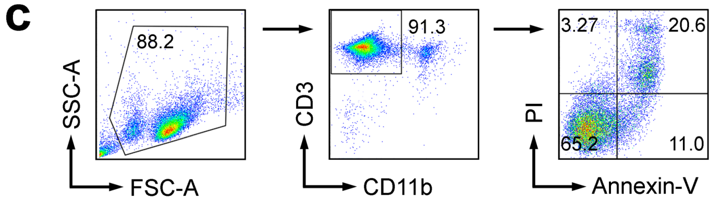

Lymphocyte apoptosis gating strategy for Fig. S3d.

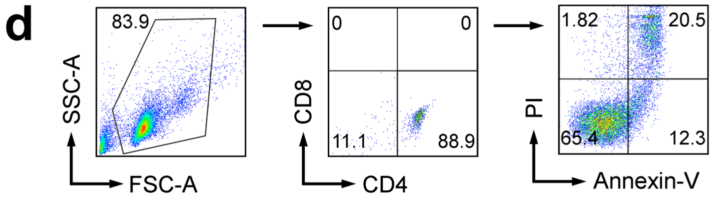

CD4+ T-cell apoptosis gating strategy for Fig. S3e.

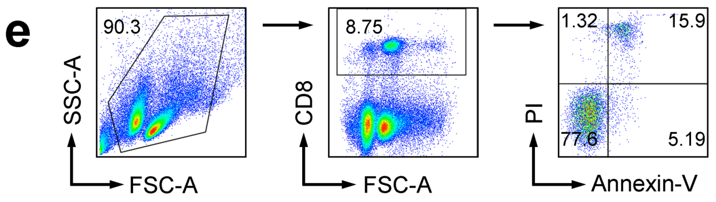

CD8+ T-cell apoptosis gating strategy for Fig. S3f.

Supplementary Fig. 4. FACS gating strategy. Gating strategies for apoptotic neutrophils (**a/b**), macrophages **(c)**, splenic lymphocytes **(d)**, CD4^{+} T cells **(e),** and CD8^{+} T cells **(f)** corresponding to the results from **Supplementary Fig. 3**.

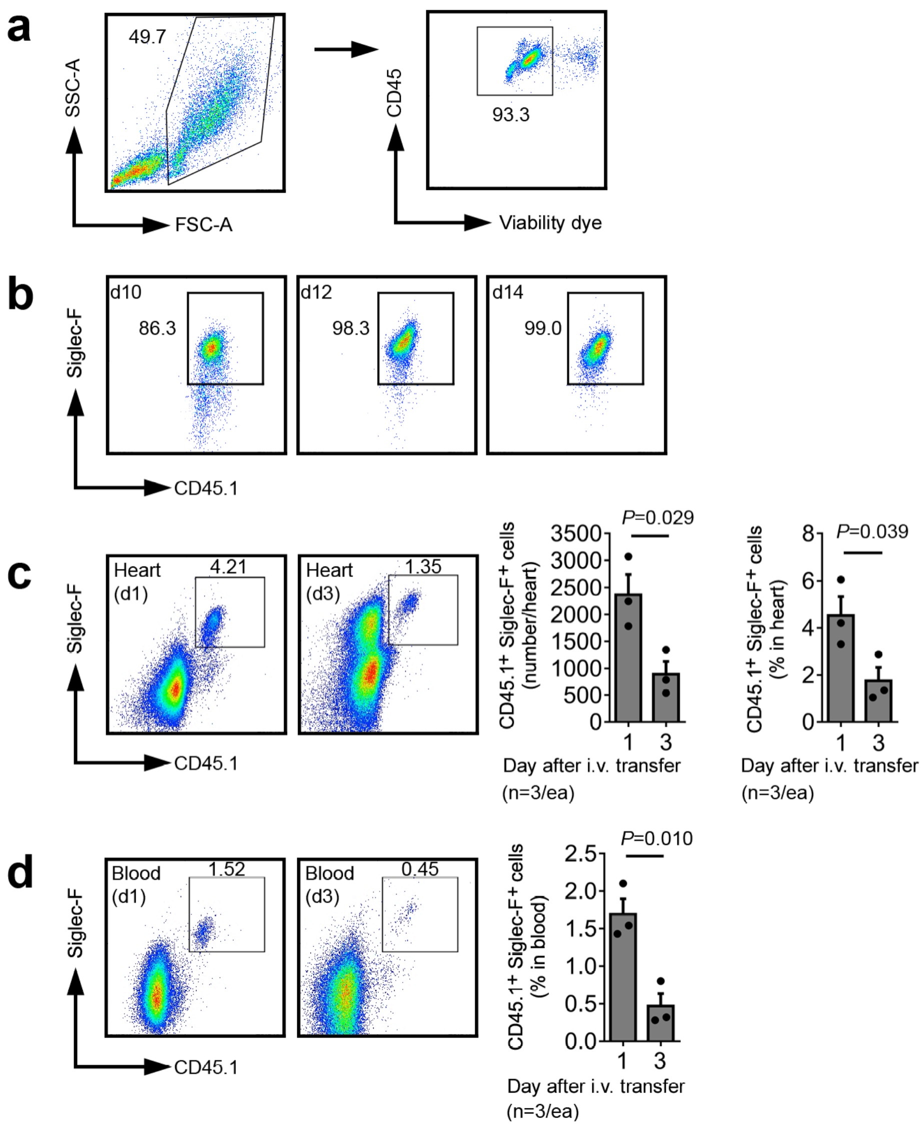

Supplementary Fig. 5. CD45.1^{+}Siglec-F^{+} EOS preparation and adoptive transfer. **a.** FACS gating strategy for purified bone marrow-derived CD45.1^{+}Siglec-F^{+} EOS. **b.** FACS determined the purity of bone marrow-derived CD45.1^{+}Siglec-F^{+} EOS at 10, 12 and 14 days after differentiation. **c.** FACS determined donor CD45.1^{+}Siglec-F^{+} EOS in heart at 1 and 3 days after i.v. adoptive transfer. **d.** FACS determined donor CD45.1^{+}Siglec-F^{+} EOS in blood at 1 and 3 days after i.v. adoptive transfer. FACS gating strategy for heart and blood CD45.1^{+}Siglec-F^{+} EOS is shown in Fig. 3h from the main text. Data are mean±SEM. The number of mice in each group and *P* values are indicated, non-parametric Mann-Whitney *U* test (**c**/**d**).

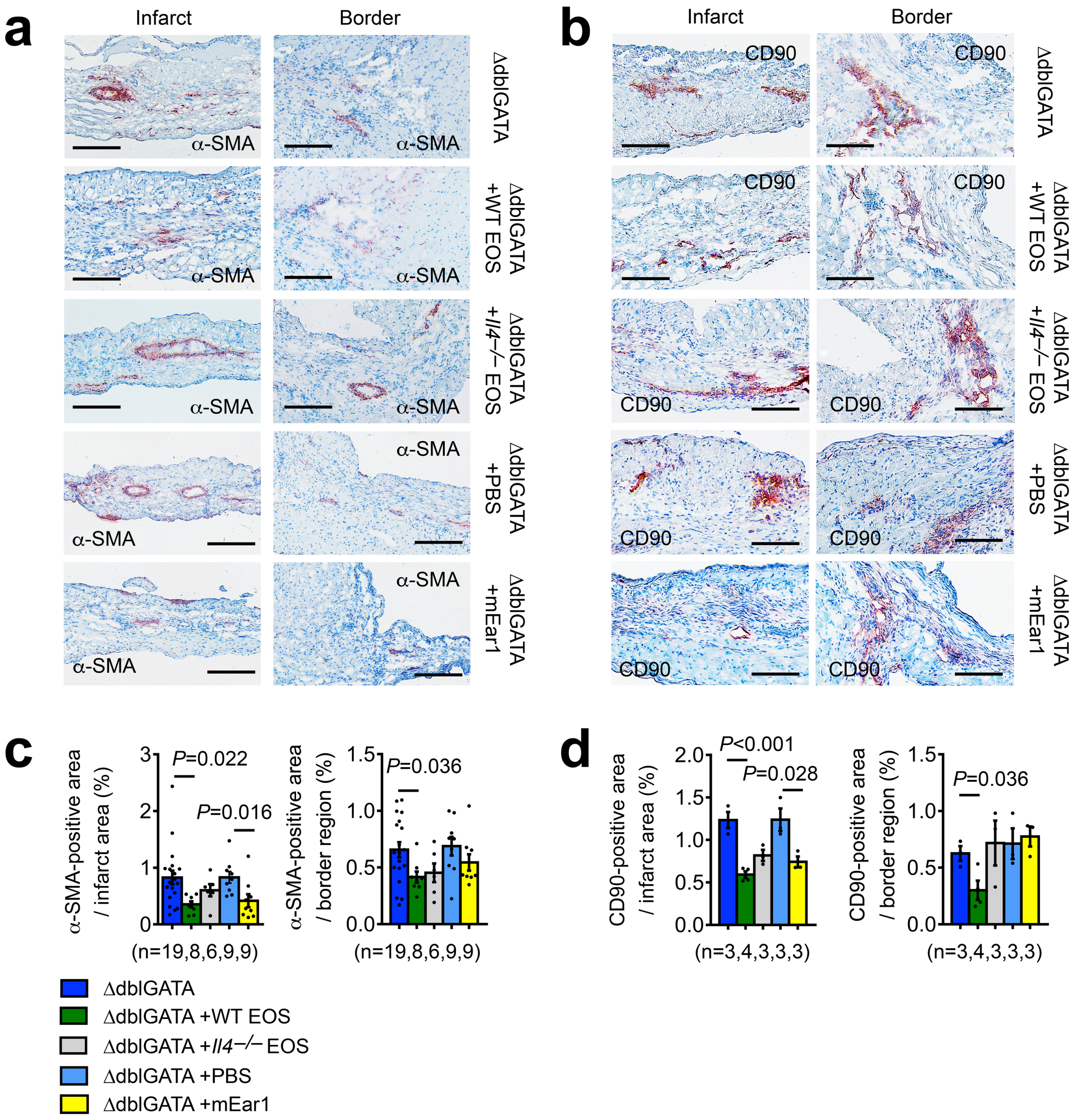

Supplementary Fig. 6. Infarct and border α-SMA- and CD90-positive areas in ΔdblGATA mice at 1-month post-MI. **a/b.** Representative images of α-SMA and CD90 staining in the infarct and border regions. **c/d.** Quantifications of α-SMA- and CD90-positive areas in the infarct and border regions from ΔdblGATA mice and those received adoptive transfers of WT EOS, *Il4*$^{-/-}$ EOS, PBS, or mEar1 (1 µg/day by minipump) at 1-month post-MI. Scale bar: 200 µm. Data are mean±SEM. The number of mice in each group and *P* values are indicated, one-way ANOVA test (**c/d**).

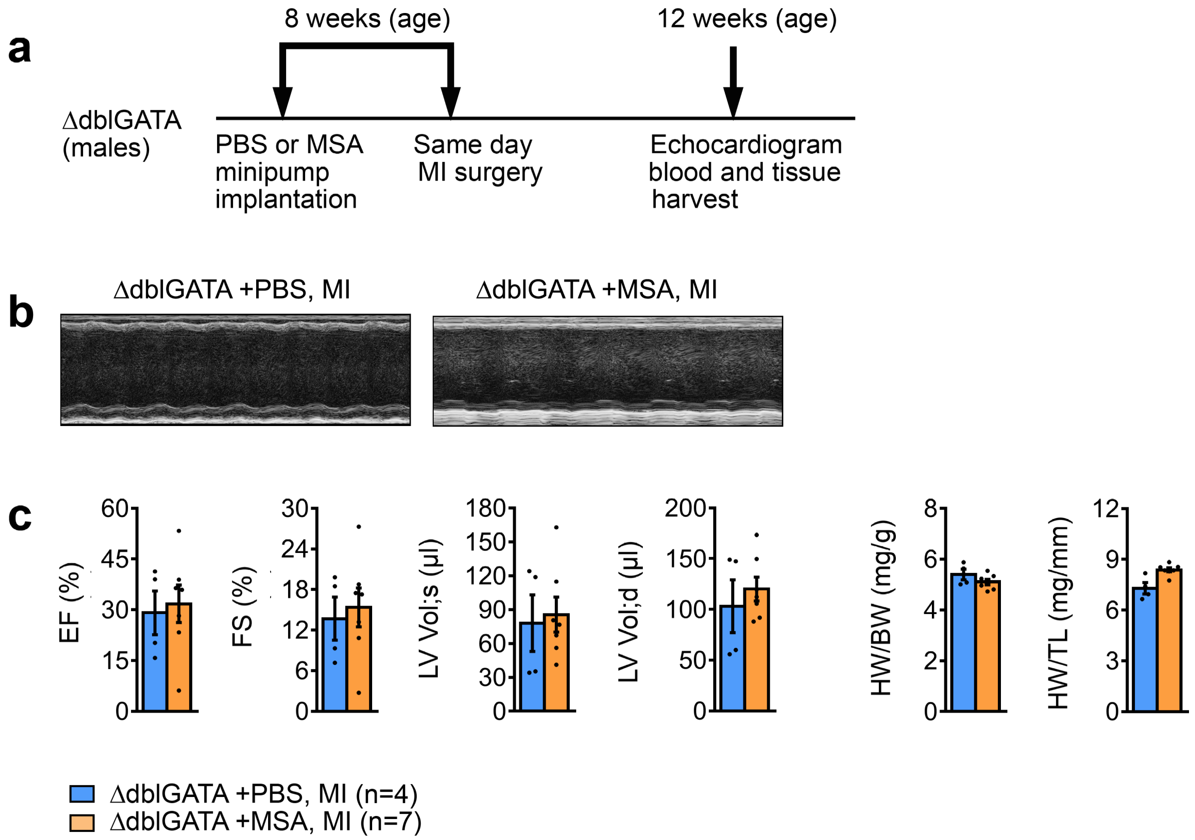

Supplementary Fig. 7. Mouse serum albumin activity in ΔdblGATA mice post-MI. **a.** MI surgery scheme. **b.** Representative LV M-mode echocardiography images at 1-monthpost-MI of ΔdblGATA mice received PBS or MSA (1 µg/day by minipump). **c.** EF, FS, LV end diastolic volume, LV end systolic volume, HW/BW, and HW/TL in ΔdblGATA mice received PBS or MSA as indicated. Data are mean±SEM. The numbers of mice in each group are indicated in the parenthesis. Non-parametric Mann-Whitney *U* test was used to test the statistical significance between the groups

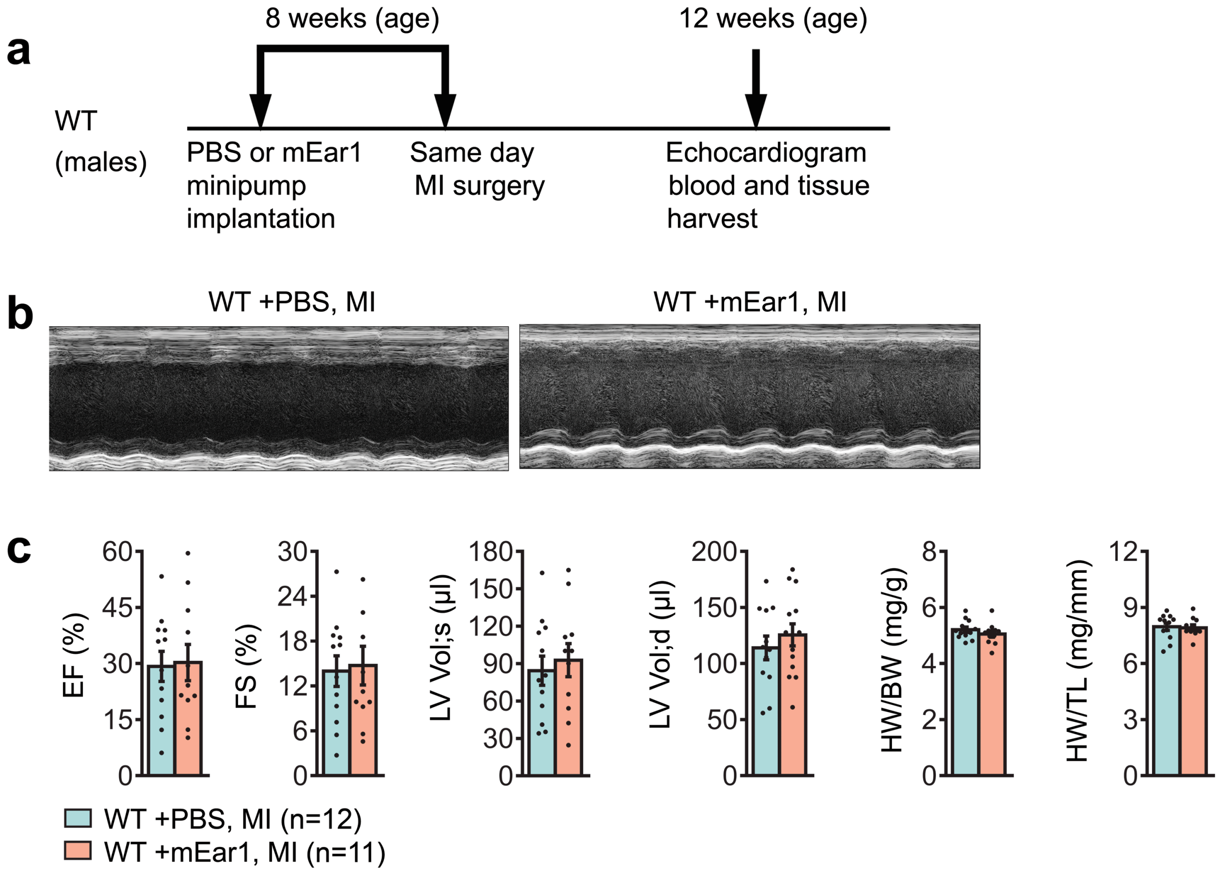

Supplementary Fig. 8. Exogenous mEar1 activity in post-MI cardiac function in WT male mice. **a.** MI surgery scheme. **b.** Representative LV M-mode echocardiography images at 1-month post-MI of male WT mice received PBS or mEar1 (1 µg/day by minipump). **c.** EF, FS, LV Vol;d, LV Vol;s, HW/BW, and HW/TL in WT mice received PBS or mEar1 as indicated. Data are mean±SEM. The numbers of mice in each group are indicated in the parenthesis. Non-parametric Mann-Whitney *U* test was used to test the statistical significance between the groups.
.

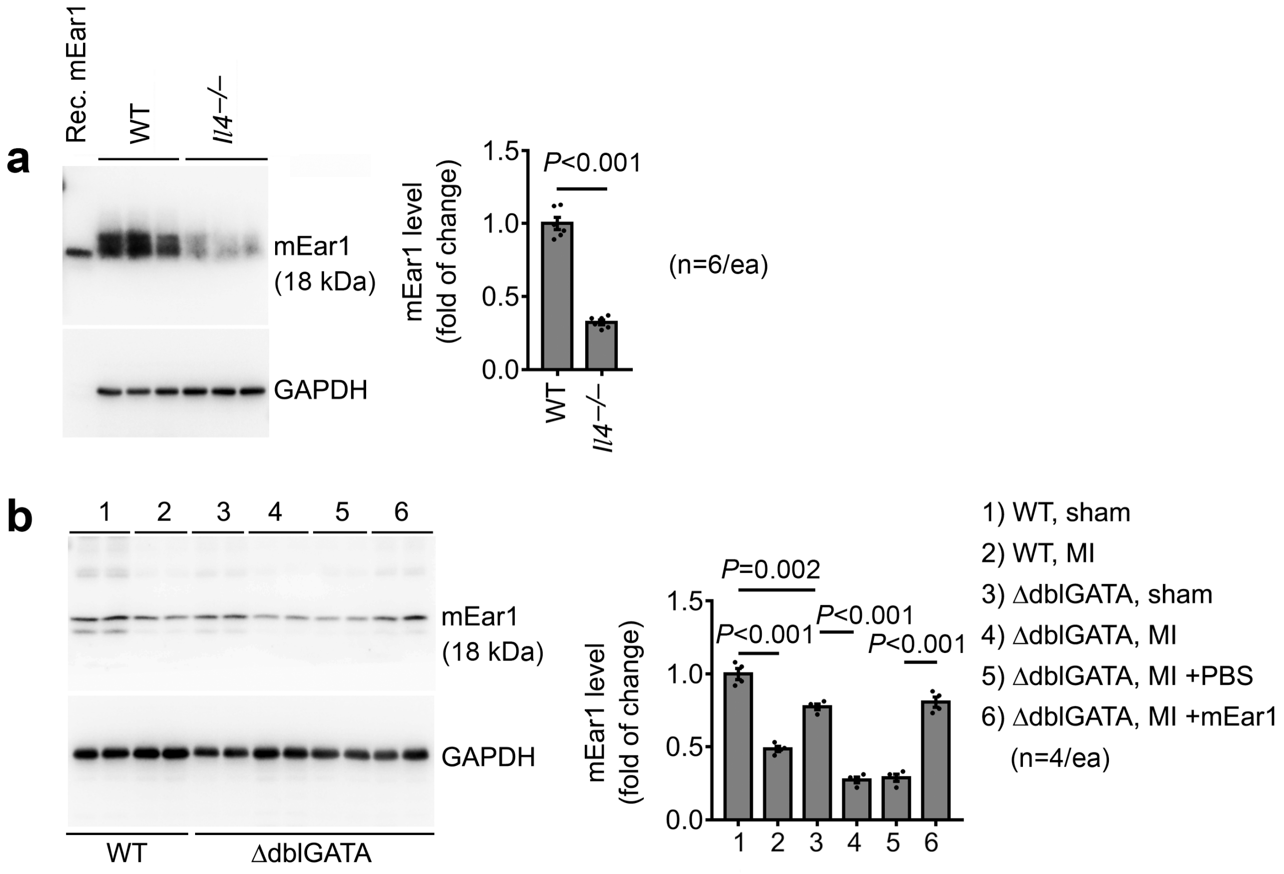

Supplementary Fig. 9. Immunoblot analysis of mEar1 expression in WT and *Il4*$^{-/-}$ EOS (**a**, recombinant mouse mEar1 was used as immunoblot size control) and in heart tissues from 1-month post-MI of different mice as indicated (**b**). Data are mean±SEM. The number of mice in each group and *P* values are indicated, non-parametric Mann-Whitney *U* test (**a**) or one-way ANOVA test (**b**).

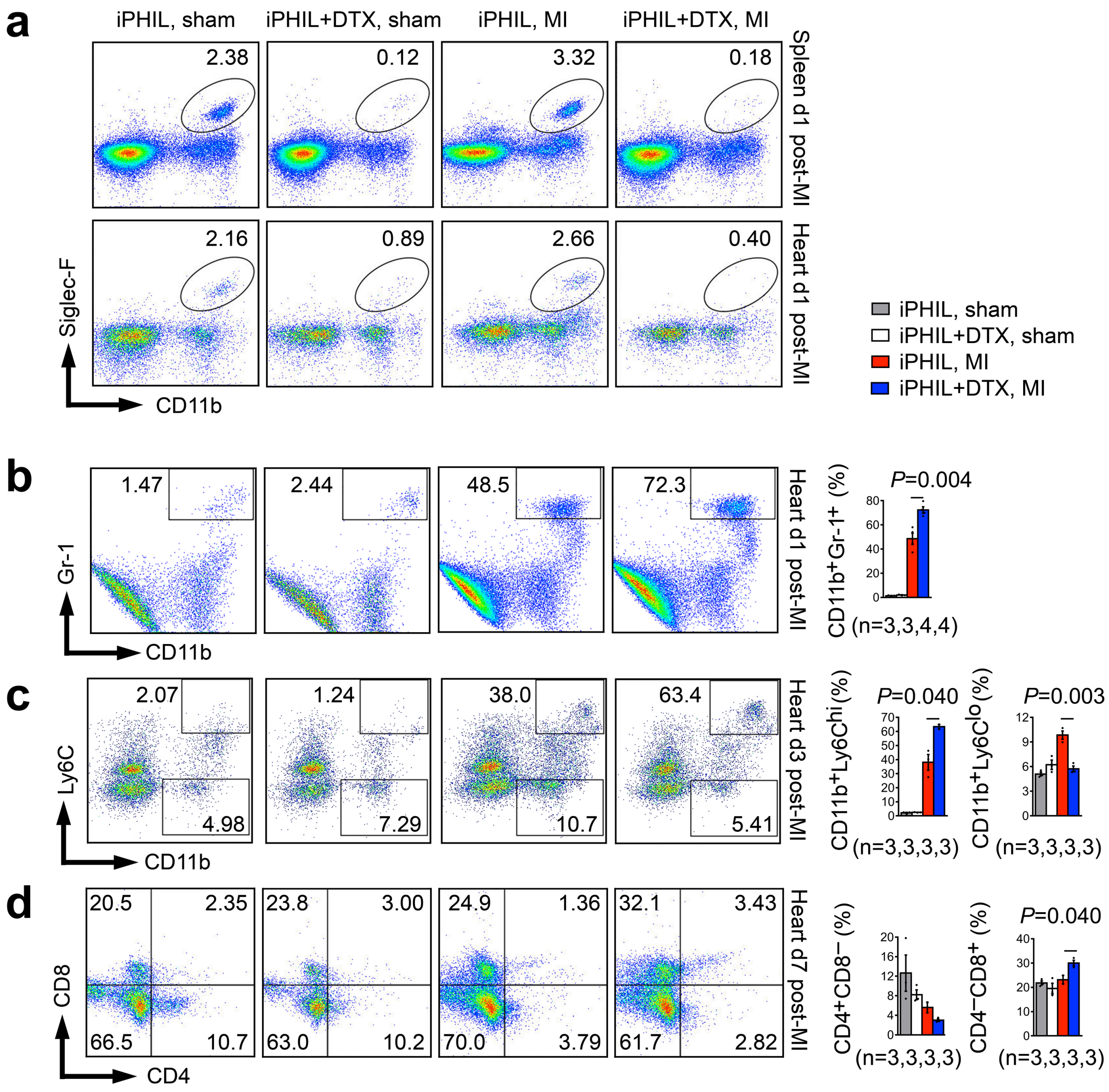

Supplementary Fig. 10. Acute heart CD45$^+$ immune cell changes post-MI in iPHIL mice with or without DTX-mediated EOS depletion. **a.** FACS ensured efficient EOS depletion in spleen and heart at 1-day post-MI. FACS detected CD11b$^+$Gr-1$^+$ neutrophils at 1-day post-MI or sham (**b**), CD11b$^+$Ly6C^{hi} and CD11b$^+$Ly6C^{lo} monocytes at 3-day post-MI or sham (**c**), and CD4$^+$CD8$^-$ and CD4$^-$CD8$^+$ T cells at 7-day post-MI or sham (**d**) operation as indicated. Representative FACS images are shown to the left. Gating strategy is shown in **Fig. 3h** from the main text. Data are mean±SEM. The number of mice in each group and *P* values are indicated, one-way ANOVA test (**b-d**).

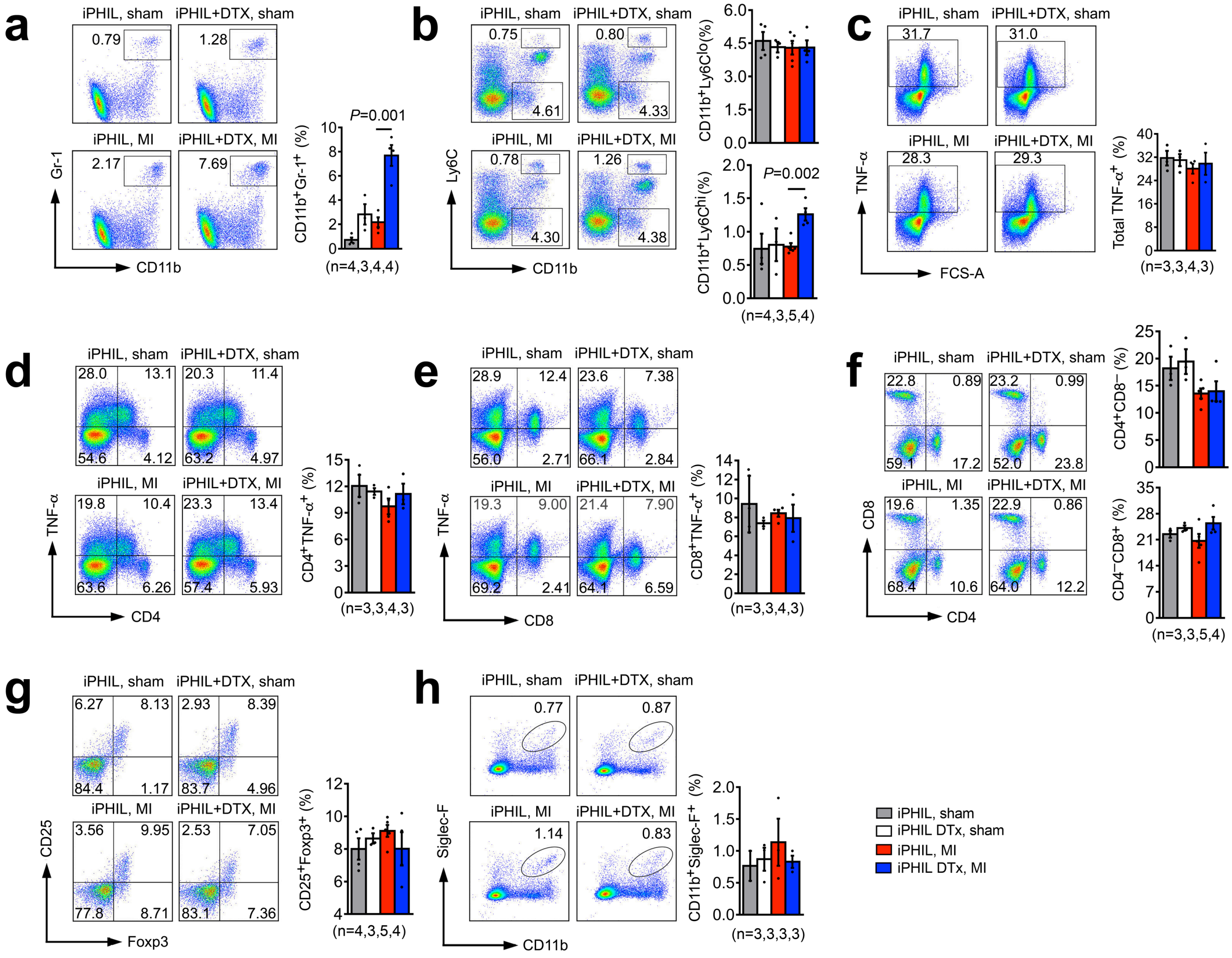

Supplementary Fig. 11. FACS analysis of splenic CD45$^+$ immune cells from different groups of mice as indicated at 1-month post-MI. **a.** CD11b$^+$Gr-1$^+$ neutrophils. **b.** CD11b$^+$Ly6C^{hi} and CD11b$^+$Ly6C^{lo} monocytes. **c.** Total TNF-α^+ cells. **d.** CD4$^+$TNF-α^+ T cells. **e.** CD8$^+$TNF-α^+ T cells. **f.** CD4$^+$CD8$^-$ and CD4$^-$CD8$^+$ T cells. **g.** CD4$^+$CD25$^+$Foxp3$^+$ Tregs. **h.** CD11b$^+$Siglec-F$^+$ EOS. Representative FACS images are shown to the left of each panel. FACS gating strategy is the same as in **Supplementary Fig. 1g**. Data are mean±SEM. The number of mice in each group and *P* values are indicated, one-way ANOVA test.

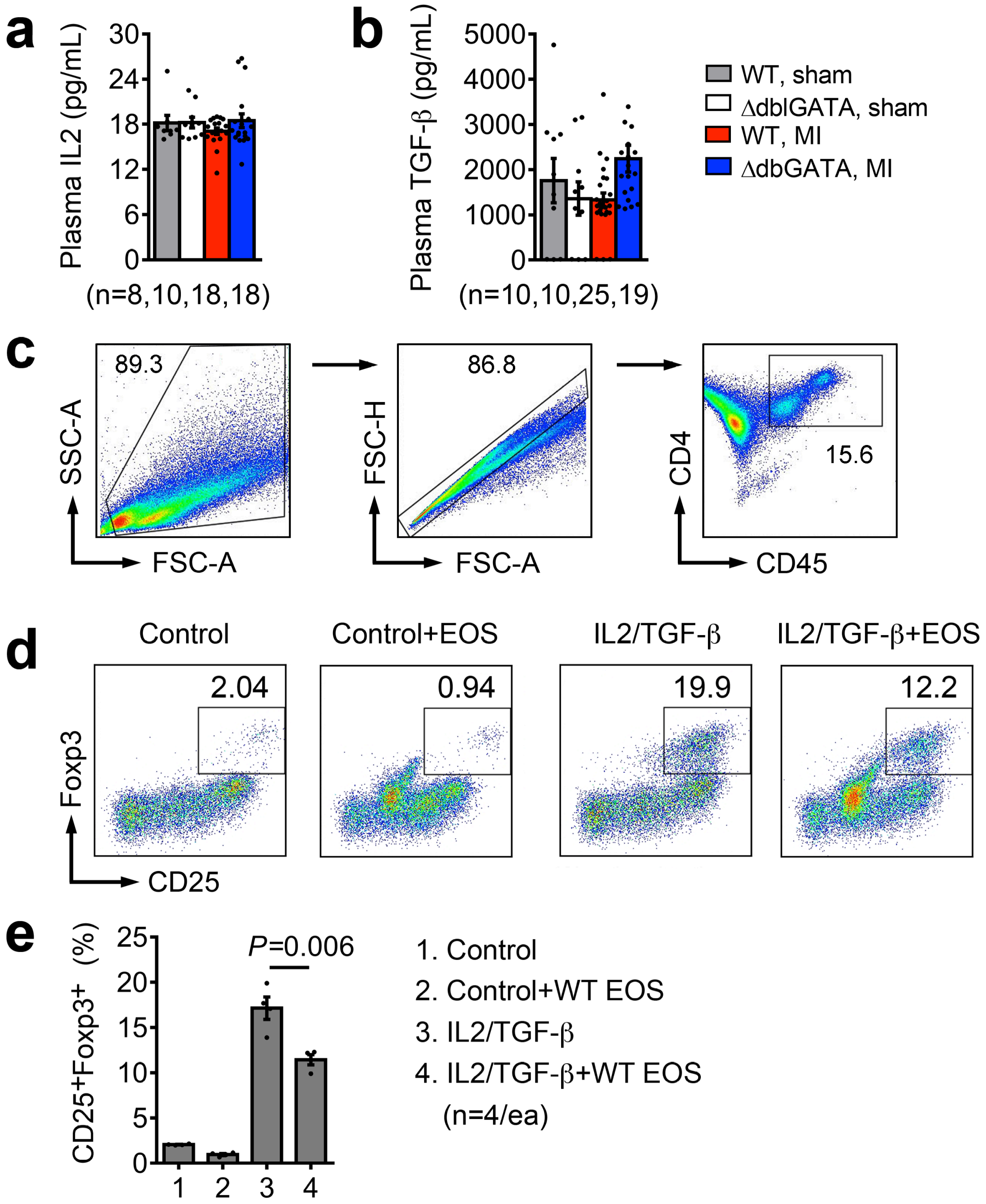

Supplementary Fig. 12. EOS reduce Treg cell differentiation. Plasma IL2 (**a**) and TGF-β (**b**) levels in WT and ΔdblGATA mice at 1-month post-MI. **c.** FACS gating strategy. **d/e.** Representative images and quantification of WT EOS activity in IL2 and TGF-β-induced $CD45^+CD4^+CD25^+Foxp3^+$ Treg cell differentiation. Data are mean±SEM. The number of mice in each group (**a/b**), the numbers of experiments in each treatment (**e**), and *P* value are indicated, one-way ANOVA test (**a/b, e**).

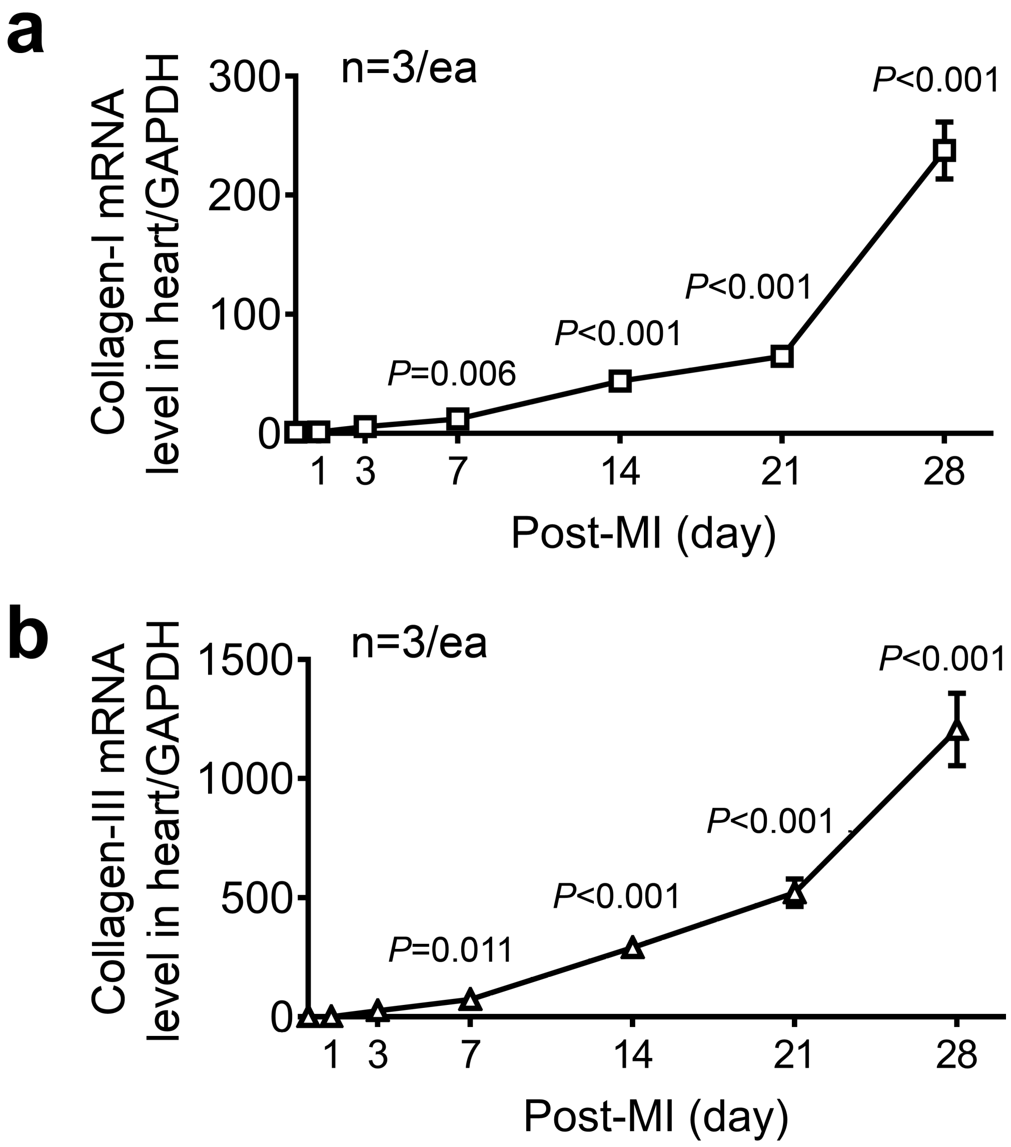

Supplementary Fig. 13. Mouse heart collagen expression post-MI. RT-PCR determined myocardium collagen-I (**a**) and collagen-III (**b**) mRNA levels in WT mice at different days post-MI. Data are mean±SEM. The number of mice in each group and *P* values compared with baseline collagen expression levels (0 day) are indicated, one-way ANOVA test (**a/b**).
