# Supplementary material for: Eosinophils improve cardiac function after myocardial infarction
Source: Nat Commun. 2020 Dec 16;11:6396. doi: 10.1038/s41467-020-19297-5 (PMC7745020; doi:10.1038/s41467-020-19297-5)
Supplement: Supplementary file 3 — Reporting Summary [file 41467_2020_19297_MOESM3_ESM.pdf]

nature research

Corresponding author(s): Guo-Ping Shi, Jing Wang, Junli Guo

Last updated by author(s): Guo-Ping Shi

Reporting Summary

Nature Research wishes to improve the reproducibility of the work that we publish. This form provides structure for consistency and transparency in reporting. For further information on Nature Research policies, see our Editorial Policies and the Editorial Policy Checklist.

Statistics

For all statistical analyses, confirm that the following items are present in the figure legend, table legend, main text, or Methods section.

n/a	Confirmed	
☐	☒	The exact sample size (n) for each experimental group/condition, given as a discrete number and unit of measurement
☐	☒	A statement on whether measurements were taken from distinct samples or whether the same sample was measured repeatedly
☐	☒	The statistical test(s) used AND whether they are one- or two-sided<br>*Only common tests should be described solely by name; describe more complex techniques in the Methods section.*
☐	☒	A description of all covariates tested
☐	☒	A description of any assumptions or corrections, such as tests of normality and adjustment for multiple comparisons
☐	☒	A full description of the statistical parameters including central tendency (e.g. means) or other basic estimates (e.g. regression coefficient) AND variation (e.g. standard deviation) or associated estimates of uncertainty (e.g. confidence intervals)
☐	☒	For null hypothesis testing, the test statistic (e.g. F, t, r) with confidence intervals, effect sizes, degrees of freedom and P value noted<br>*Give P values as exact values whenever suitable.*
☒	☐	For Bayesian analysis, information on the choice of priors and Markov chain Monte Carlo settings
☒	☐	For hierarchical and complex designs, identification of the appropriate level for tests and full reporting of outcomes
☐	☒	Estimates of effect sizes (e.g. Cohen's d, Pearson's r), indicating how they were calculated

Our web collection on statistics for biologists contains articles on many of the points above.

Software and code

Policy information about availability of computer code

Data collection	For mouse cardiac function, we used Vevo3100 for ultrasound and used Vevo Lab v3.2.0 software for related analyses. We have strain analysis and 3D tools to analyze real-time functional and structural data. For flow cytometry, we used the BD FACSCanto II to collect the data and used Flowjo 10.4.1 for related analyses. For western blot, we used Image Lab 6.0 for related analyses.
Data analysis	SPSS 20.0 and GraphPad Prism 8.2.0 were used in this study

For manuscripts utilizing custom algorithms or software that are central to the research but not yet described in published literature, software must be made available to editors and reviewers. We strongly encourage code deposition in a community repository (e.g. GitHub). See the Nature Research guidelines for submitting code & software for further information.

Data

Policy information about availability of data

All manuscripts must include a data availability statement. This statement should provide the following information, where applicable:

- Accession codes, unique identifiers, or web links for publicly available datasets
- A list of figures that have associated raw data
- A description of any restrictions on data availability

All raw data are available upon request.

Field-specific reporting

Please select the one below that is the best fit for your research. If you are not sure, read the appropriate sections before making your selection.

☒ Life sciences ☐ Behavioural & social sciences ☐ Ecological, evolutionary & environmental sciences

For a reference copy of the document with all sections, see nature.com/documents/nr-reporting-summary-flat.pdf

Life sciences study design

All studies must disclose on these points even when the disclosure is negative.

Sample size	Sample sizes were assessed using the power calculation at p<0.05, power >0.80.
Data exclusions	No data were excluded from the analyses.
Replication	Each independent experiment was replicated at least 3-4 times. All the attempts at replication were successful.
Randomization	All mice and tissue samples grouped blindly based on mice/sample availability.
Blinding	The investigators were blinded to group allocation during data collection and analysis.

Reporting for specific materials, systems and methods

We require information from authors about some types of materials, experimental systems and methods used in many studies. Here, indicate whether each material, system or method listed is relevant to your study. If you are not sure if a list item applies to your research, read the appropriate section before selecting a response.

Materials & experimental systems

n/a	Involved in the study	
☐	☒	Antibodies
☒	☐	Eukaryotic cell lines
☒	☐	Palaeontology and archaeology
☐	☒	Animals and other organisms
☐	☒	Human research participants
☐	☒	Clinical data
☒	☐	Dual use research of concern

Methods

n/a	Involved in the study	
☒	☐	ChIP-seq
☐	☒	Flow cytometry
☒	☐	MRI-based neuroimaging

Antibodies

Antibodies used

Viability Dye (Cat#65-0866-14 and Cat#65-0863-14), CD45 (Cat#25-0451-82 and Cat#11-0451-85), CD45.1 (Cat#45-0453-82), CD11b (Cat#17-0112-82), Siglec-F (Cat#12-1702-82), Gr-1 (Cat#12-5931-82), Ly6C (Cat#53-5932-82), CD11c (Cat#53-0114-82), CD4 (Cat#A15384), CD8 (Cat#12-0081-82), CD25 (Cat#48-0253-80), Foxp3 Transcription Factor Staining Kits (Cat#A25864A) and TNF--Alexa Fluor 488 (Cat#53-7321-82) are all from eBioscience, San Diego, CA. CCR3 (Cat#144516) and MHC-II (Cat#107607) are from BioLegend, San Diego, CA.
PECAM-1 (Cat#553389) and ICAM-2 (Cat#553325) are from BD Biosciences, Bedford, MA.
Rabbit anti-mouse mEar1 polyclonal antibody, Cat#orb13385, Biorbyt, San Francisco, CA.
Rabbit anti-mouse total Smad-2 (Cat#5339S), rabbit anti-mouse p-Smad-2 (Cat#3108S), rabbit anti-mouse total Smad-3 (Cat#9523S) and and rabbit anti-mouse GAPDH (Cat#2118S) are all from Cell Signaling Technology, Beverly, MA.
Rabbit anti-mouse p-Smad-3, Cat#ab52903, Abcam, Cambridge, MA.
Rat anti-mouse CD3 monoclonal antibodies, Cat#100223, BioLegend, San Diego, CA.
Ramster anti-mouse CD28 monoclonal antibodies, Cat#102121, BioLegend, San Diego, CA.
Rabbit anti-mouse GFP antibody, Cat#ab183734, Abcam, Cambridge, MA.
Rabbit anti-mouse cleaved caspase-3 monoclonal antibody, Cat#8172, Cell Signaling Technology, Beverly, MA.
Mouse anti-mouse-SMA monoclonal, Cat#F3777, Sigma-Aldrich, Louis, MO.
Rabbit anti-mouse cardiac myosin heavy chain (MYH) polyclonal antibody, Cat#bs-15444R-A488, Bioss Inc., Woburn, MA.
Rabbit anti-mouse Bcl2 monoclonal antibody, Cat #3498S, Cell Signaling Technology, Beverly, MA

Validation

All FACS antibodies used in this study, including Viability Dye (Cat#65-0866-14 and Cat#65-0863-14), CD45 (Cat#25-0451-82 and Cat#11-0451-85), CD45.1 (Cat#45-0453-82), CD11b (Cat#17-0112-82), Siglec-F (Cat#12-1702-82), Gr-1 (Cat#12-5931-82), Ly6C (Cat#53-5932-82), CD11c (Cat#53-0114-82), CD4 (Cat#A15384), CD8 (Cat#12-0081-82), CD25 (Cat#48-0253-80), Foxp3 Transcription Factor Staining Kits (Cat#A25864A) and TNF--Alexa Fluor 488 (Cat#53-7321-82) were validated by using mouse spleenocytes. And all of them worked well in this study.
PECAM-1 (Cat#553389) and ICAM-2 (Cat#553325) were validated by using mouse endothelial cells. Rabbit anti-mouse mEar1

	polyclonal antibody (Cat#orb13385) was validated by using mouse plasma. Rabbit anti-mouse total Smad-2 (Cat#5339S), rabbit anti-mouse p-Smad-2 (Cat#3108S), rabbit anti-mouse total Smad-3 (Cat#9523S), rabbit anti-mouse GAPDH (Cat#2118S) and Rabbit anti-mouse p-Smad-3 (Cat#ab52903) were validated by using mouse cardiac fibroblasts. Rat anti-mouse CD3 monoclonal antibodies, (Cat#100223) and Ramster anti-mouse CD28 monoclonal antibodies (Cat#102121) were validated by using mouse spleenocytes. Rabbit anti-mouse GFP antibody (Cat#ab183734) was validated by using GFP mouse heart tissue. Rabbit anti-mouse cleaved caspase-3 monoclonal antibody (Cat#8172) was validated by using mouse cardiomyocytes. Mouse anti-mouse-SMA (Cat#F3777) and Rabbit anti-mouse cardiac myosin heavy chain (MYH) polyclonal antibody (Cat#bs-15444R-A488) were validated by using mouse heart tissue.

Animals and other organisms

Policy information about studies involving animals; ARRIVE guidelines recommended for reporting animal research

Laboratory animals	Male or female Il4–/– (002253), Il10–/– (002251) and Il13–/– micw, 45.1 transgenic mice (002014), C57BL/6 mice (000664), male and female ΔdblGATA (005653), and Balb/c (000651) mice aged 7 to 8 weeks were purchased from the Jackson laboratory. eoCRE and EOS-less iPHIL mice were provided by Mayo clinic. eoCRE mice were crossed with a (flox-stop-flox)-GFP reporter strain (B6.Cg-Gt (ROSA)26Sortm6(CAG-ZsGreen1)Hze/J, 007906) purchased from the Jackson laboratory. All of those mice were housed in a specific pathogen-free facility on a 12 light/12 dark cycle at temperatures of 65-75°F with 40-60% humidity.<br>We also cultured eosinophils from C57BL/6 wild type mice, Balb/c wild-type mice, CD45.1 mutant allele mice, and Il4–/–, Il10–/–, Il13–/– mice. We isolated cardiomypocytes and fibroblasts from C57BL/6 wild type mice. We also isolated splenocytes, bone marrow neutrophils and cultured bone marrow derived macrophages from C57BL/6 wild type mice.
Wild animals	We used C57BL/6 (000664) and Balb/c (000651) wild type mice purchased from the Jackson laboratory. No wild animals and no field collected samples were used in the study.
Field-collected samples	We collected mouse heart to test the heart immune cells in different time points post-MI, such as post-MI day 1, 3, 5, and 7. We collected mouse heart for immunostainning one-month post-MI. We also collected spleen for immune cell analysis. We collected liver, kidney, plasma for potential use. No field collected samples were used in the study.
Ethics oversight	All animal procedures conformed to the guide for the Care and Use of Laboratory Animal published by the US National Instituted of Health and was approved by the Brigham and Women's Hospital Standing Committee on Animals (protocol #2016N000442)

Note that full information on the approval of the study protocol must also be provided in the manuscript.

Human research participants

Policy information about studies involving human research participants

Population characteristics	The DANCAVAS trial is a population-based, randomized, and clinically-controlled screening trial of men aged 65-74. No exclusion criteria were used. One-third was invited for cardiovascular screening examinations including a CT scan at one of the four locations, among which 62.4% men attended. The screening includes a low-dose, noncontrast computerized tomography scan to detect coronary artery calcification and aortic and iliac aneurysms; brachial and ankle blood pressure index to detect peripheral arterial disease and hypertension; a telemetric assessment of heart rhythm; and a measurement of blood cholesterol and glucose levels. For the purpose of this study, leucocyte count, including EOS, was also performed. At attendance of screening, each man was first given the informed consent, then a medical history was obtained including previous acute myocardial infarction (AMI), coronary revascularization, chronic obstructive pulmonary disease (COPD), medication history, and symptoms. In case of dyspnoea, NYHA classification was performed. Up-to-date cardiovascular preventive treatment was recommended in case of subclinical cardiovascular disease. Use of decoded patient information was pre-proved by the Human Investigation Review Committee at the Brigham and Women's Hospital, Boston, MA, USA (protocol #2010P001930). No patient informed consent was required.<br><br>Human blood was from healthy donor in Massachusetts General Hospital.
Recruitment	The human population included men attending the Danish Cardiovascular Screening Trial (DANCAVAS) in Odense. We consecutively selected 5,864 men who had blood EOS counts available. The DANCAVAS trial is a population-based, randomized, and clinically-controlled screening trial of men aged 65-74. No exclusion criteria were used. One-third was invited for cardiovascular screening examinations including a CT scan at one of the four locations, among which 62.4% men attended. The screening includes a low-dose, noncontrast computerized tomography scan to detect coronary artery calcification and aortic and iliac aneurysms; brachial and ankle blood pressure index to detect peripheral arterial disease and hypertension; a telemetric assessment of heart rhythm; and a measurement of blood cholesterol and glucose levels. For the purpose of this study, leucocyte count, including EOS, was also performed. At attendance of screening, each man was first given the informed consent, then a medical history was obtained including previous acute myocardial infarction (AMI), coronary revascularization, chronic obstructive pulmonary disease (COPD), medication history, and symptoms. In case of dyspnoea, NYHA classification was performed. Up-to-date cardiovascular preventive treatment was recommended in case of subclinical cardiovascular disease.
Ethics oversight	Use of decoded patient information was pre-proved by the Human Investigation Review Committee at the Brigham and Women's Hospital, Boston, MA, USA (protocol #2010P001930). No patient informed consent was required.

Note that full information on the approval of the study protocol must also be provided in the manuscript.

Clinical data

Policy information about clinical studies

All manuscripts should comply with the ICMJE guidelines for publication of clinical research and a completed CONSORT checklist must be included with all submissions.

Clinical trial registration	N/A
Study protocol	2010P001930
Data collection	We consecutively selected 5,864 men who had their blood samples taken and blood EOS counts available (from January 2015 to August 2018). Of these, 345 men had suffered from an acute MI (AMI). We collected the baseline characteristics. Such as body mass index, current or former smokers, diabetes, ischaemic heart disease, chronic obstructive pulmonary disease, use of low-dose aspirin, statins, loop diuretics, and inhalation therapy associated significantly with high blood EOS counts.
Outcomes	we consecutively selected 5,864 men who had their blood samples taken and blood EOS counts available (from January 2015 to August 2018). Of these, 345 men had suffered from an acute MI (AMI). Men with previous AMI had significantly higher blood EOS counts than those without AMI. Multivariate logistic regression analysis suggests that high blood EOS counts posed a significant risk factor of human AMI, and such significance persisted after adjustment for potential confounders. The BMI group, former or current smoking, and use of statins and low-dose aspirin also significantly associated with EOS counts, while data from COPD and diabetics did not reveal any significance. High blood EOS counts associated positively with the Eur-Qol-5D and NYHA classifications. Higher EOS counts associated with lower mobility. The association with the NYHA classification group remained significant after adjustment. In a subgroup of 482 men from our cohort who underwent echocardiography for other research purposes, blood EOS counts correlated significantly and negatively with the ejection fraction (EF, r=-0.122, P=0.013). This association significance became weaker but persisted after adjustment of the potential confounders (partial correlation coefficient r=-0.094, P=0.037).

Flow Cytometry

Plots

Confirm that:

- [x] The axis labels state the marker and fluorochrome used (e.g. CD4-FITC).
- [x] The axis scales are clearly visible. Include numbers along axes only for bottom left plot of group (a 'group' is an analysis of identical markers).
- [x] All plots are contour plots with outliers or pseudocolor plots.
- [x] A numerical value for number of cells or percentage (with statistics) is provided.

Methodology

Sample preparation	Heart total single cell preparation. The heart was perfused with 20 ml of cold PBS and then removed from mouse. Heart tissue was minced into small pieces and digested in a 0.1% collagenase B (LS004177, Worthington Biochemical Co, Lakewood, NJ) dissolved in a HEPES buffer for 30 min in a 37 °C water bath. Vortex was performed every 10 min. After digestion, cells were neutralized using 1640 culture medium supplemented with 10% fetal bovine serum (FBS) and washed twice. Cells were then separated using density gradient centrifugation in a 15-ml tube layered with 2 ml 100% Percoll (17-0891-09, Fisher Scientific, Hampton, NH), 1.5 ml 80% Percoll, 1.5 ml 62% Percoll, 1.5 ml 55% Percoll, and 3 ml 45% Percoll that was pre-mixed with the cell preparation. Cells were centrifuged at 800 g for 30 min. FACS were performed to measure eosinophils, neutrophil, Ly6Chi and Ly6Clo monocytes, dendritic cells (DCs), CD4+ and CD8+ T cells in infarcted heart.<br><br>Splenocyte isolation. The spleen was removed from the mouse, placed in a cold PBS, and grinded in 5 ml PBS before filtering through a 70-μm cell strainer. Splenocytes were collected after depleting the red blood cells using the rec blood cell lysis buffer (004333). Splenocytes were then stained with cell viability dye and antibodies against CD45, CD11b, Gr-1, CD4, CD8, and CD25 (48-0253-80) (all from eBioscience). To analyze TNF-+ cells, splenocytes were immunostained with CD4 and CD8 first, and then stimulated with a Cell Stimulation Cocktail (00-4975-03, eBioscience) at 37 °C for 5 hours in the dark to induce intracellular cytokine expression and accumulation before intracellular staining was performed. Cells were fixated with the IC Fixation Buffer (00-8222-49), permeabilized with the permeabilization buffer (00-8333-56), and immunostained with rat anti-mouse TNF--Alexa Fluor 488 (53-7321-82). For the detection of T-regulatory cells, the cells were incubated with anti-mouse CD4 and CD25 antibodies at 4°C for 30 min in the dark to perform cell surface maker staining. Cells were then fixated, permeabilized, and stained with the anti-mouse Foxp3 antibody using the Foxp3 Transcription Factor Staining Buffer Kit according to the manufacturer's instructions (A25864A).
Instrument	We used BD FACSCanto II for data collection.
Software	We used flowjo 10.4.1 to analyze the data.
Cell population abundance	No sorting was used in this manuscript.
Gating strategy	First, immune cells were gated on the SSC/FSC plots, then single cells were gated on the FSH/FSC plots. Interested cells were stained with different marker. Gating was determined by blank and single color-staining.

- [x] Tick this box to confirm that a figure exemplifying the gating strategy is provided in the Supplementary Information.
